# Workshop report: “Towards a Cure: HIV Reservoirs and Strategies to Control Them”

**DOI:** 10.1186/1758-2652-13-S3-I1

**Published:** 2010-11-04

**Authors:** Richard J Jefferys

**Affiliations:** 1 Michael Palm Basic Science, Vaccines & Prevention Project, Treatment Action Group, 611 Broadway, Suite 308, New York, NY 10012, USA

## Abstract

On 16 and 17 July 2010, immediately prior to the XVIII International AIDS Conference in Vienna, Austria, the International AIDS Society held a workshop on the important topic of moving beyond antiretroviral therapy and addressing HIV persistence. “Towards a Cure: HIV Reservoirs and Strategies to Control Them” was chaired by Nobel laureate Françoise Barré-Sinoussi and co-sponsored by the French National Agency for Research on AIDS and Viral Hepatitis, Bundesministerium für Wissenschaft und Forschung, the National Institutes of Health, Sidaction and the Treatment Action Group. This article gives an overview of the findings presented at the workshop; complete abstracts are included in this supplement to the *Journal of the International AIDS Society*.

## Introduction

The availability of antiretroviral therapies (ART) capable of prolonged suppression of HIV replication has dramatically altered the AIDS research landscape. The aim of the workshop, “Towards a Cure: HIV Reservoirs and Strategies to Control Them”, was to invigorate efforts to move beyond ART, either by eradicating the virus (sterilizing cure) or by achieving long-term remission in the absence of ongoing therapy (functional cure). The organizers placed a particular focus on encouraging young investigators to work on this critically important topic, with the secondary aim of improving the breadth and quality of scientific presentations in the biomedical and pre-clinical field at the XVIII International AIDS Conference (abstracts for the workshop were selected from submissions to the conference).

Workshop attendees represented a broad array of stakeholders, including basic and clinical researchers, research funders, policymakers, community advocates and journalists. The agenda over the two days was apportioned into discrete sections, each addressing a different sub-topic; these divisions are mirrored in the subheads of this report.

## Main text

### Clinical implications of HIV persistence during therapy

Steven Deeks from the University of California at San Francisco (UCSF), USA, opened the meeting with a clinical perspective on HIV persistence. Deeks cited evidence that more than 80% of individuals on suppressive ART have persistent low-level viremia [1]. Levels of inflammatory biomarkers are also higher in treated HIV-positive people compared with their HIV-negative counterparts [2] and linked to an increased risk of morbidity and mortality [3]. Deeks described how the gut is a key site of HIV persistence, with levels of HIV DNA approximately three to nine times higher than other locations in the body [4]; a correlation has also been reported between the size of the viral reservoir in the gut and the proportion of activated CD8 T cells [5].

Taken together, Deeks noted, these data raise the question of whether persistent HIV replication causes immune activation and inflammation. However, several studies have been conducted involving intensification of antiretroviral therapy, and in all but one case [6], there was no measurable impact on persistent viremia or markers of immune activation [7-9]. Deeks’s conclusion is that “the simple pathway probably is not happening”. Further evidence comes from looking at the specific example of vascular health as a surrogate for “non-AIDS” morbidities, such as cardiovascular disease; Deeks showed data from his colleague, Hiroyu Hatano, indicating no correlation between HIV DNA or RNA levels and vascular dysfunction. In contrast, correlations between T cell activation and vascular dysfunction have been reported [10].

Deeks then reversed his previous question, and asked whether inflammation and immune activation contribute to HIV persistence. In this case, he suggested the answer is yes: there is published evidence that lower CD4 counts are associated with increased T cell turnover and higher frequencies of infected cells; this type of T cell proliferation is referred to as “homeostatic” [11]. Data also suggest that immune activation provides more activated CD4 T cell targets for HIV [12].

Deeks closed his talk with some preliminary results from his group showing that higher levels of HIV-specific CD8 T cell responses in the gut are associated with lower levels of HIV DNA, perhaps indicating that bolstering antiviral immune responses could be one way of interrupting the ongoing cycle of immune activation and viral infection that sustains the HIV reservoir.

### Where and what are viral reservoirs? HIV-1 reservoirs and sanctuary sites

Satya Dandekar from the University of California at Davis, USA, provided an overview talk on the location of HIV reservoirs in the body. Dandekar highlighted two overarching issues: the types of cells that are infected; and their anatomic locations. The major cell types include CD4 T cells, monocyte/macrophages, hematopoietic progenitor cells and microglial cells; the gut and brain represent the main anatomic locations. Dandekar noted that the gut and brain share certain features, including inaccessibility to drugs and increased numbers of activated immune cells. Importantly, HIV reservoirs in these locations are established early in primary infection. Dandekar suggested that the SIV/macaque model can be used to address a number of questions relating to viral persistence, including the role of early ART, the effect of strategies to improve gut mucosal integrity, and the efficacy of approaches designed to enhance virus-specific immunity, such as therapeutic vaccines.

**Figure 1:**
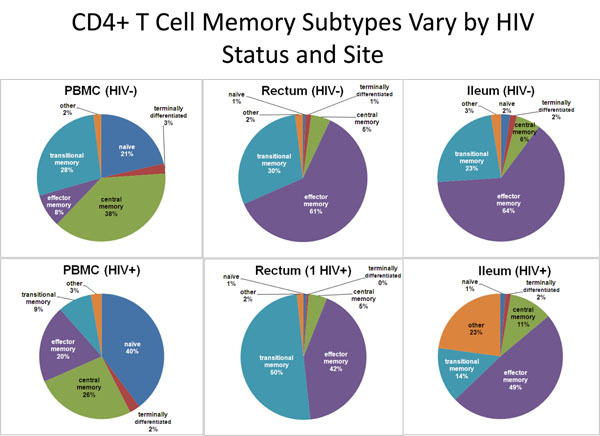
**The others in the ileum.** Joseph Wong identified an unusual subset of CD45RO+ CCR7+ CD27- “other memory” CD4 T cells in the ileum of HIV-positive individuals that may be linked to cryptic viral replication in this compartment. This slide was presented by Joseph Wong at the workshop “Towards a Cure: HIV Reservoirs and Strategies to Control Them”.

Five oral abstracts were presented in this workshop session; in concordance with Dandekar’s talk, three focused on HIV reservoirs in the gut, while the other two evaluated infection in the brain. Joseph Wong from UCSF showed data indicating that the activation status and the proportions of different memory CD4 T cell subsets varies across gut sites, and this may explain his group’s finding that the terminal ileum is a site of ongoing viral replication in individuals lacking detectable HIV RNA elsewhere (Figure 1) [4,13].

John Zaunders from St Vincent’s Hospital in Sydney, Australia, studied peripheral blood CD4 T cells in order to assess whether expression of the gut-homing marker, integrin ß7, was associated with higher levels of infection (measured by HIV DNA). While HIV DNA could be found in some CD4 T cells expressing ß7, the majority of infected cells did not express the marker. Zaunders concluded that most HIV DNA-containing CD4 T cells in the peripheral blood were not activated and infected in the gut [14].

Melissa Churchill from the Burnet Institute in Melbourne, Australia, measured the extent of astrocyte infection in the post-mortem brain tissue of individuals with varying histories of HIV-associated encephalitis (HIVE) and/or dementia (HAD). Churchill noted that astrocyte infection has been described as rare in the literature [15] and is also the subject of some controversy. Combining laser capture and single-cell microdissection with sensitive Alu-PCR, Churchill was able to find integrated HIV-DNA in astrocytes and uncovered a significant correlation with both HAD and the severity of HIVE. In some cases, close to 20% of astrocytes in the deep white matter were infected with HIV, significantly more than in previous reports.

Ronald Swanstrom from the University of North Carolina at Chapel Hill, USA, highlighted the appearance of macrophage-tropic viruses at late stages of the disease, which are compartmentalized in the central nervous system [16]. Swanstrom posited that the emergence of these viruses may be another example of HIV expanding its host cell range over the course of infection.

### What are the mechanisms of persistence?

Eric Verdin from UCSF led the workshop session on the molecular mechanisms involved in HIV latency and persistence. Verdin reviewed an array of mechanisms that can silence the transcription of HIV DNA leading to the establishment of latency. The process is multifactorial and involves the absence of key transcription factors (e.g., NF-kB, NF-AT, STAT5, P-TEFb), recruitment of chromatin modifiers (e.g., HDACs) and DNA methylation. All of these factors can conspire to repress HIV DNA transcription.

Verdin noted that latency is likely to be heterogeneous because different factors can act at different sites of viral integration, and this suggests that therapeutic strategies aiming to reduce the HIV reservoir will need to combine multiple different approaches. However, methylation of the HIV genome is common in a large fraction of latently infected cells, and inhibitors of methylation potently synergize with trans-acting factors, such as TNF and prostratin, to reactivate latent HIV *in vitro*. Verdin closed by suggesting that future efforts will need to focus more on mechanisms of latency in primary lymphoid cells taken directly from HIV-positive individuals and/or in model systems that better replicate the *in vivo* situation than standard cell lines. Three presentations followed that described different factors involved in the regulation of latency, including CTIP2 and P-TEFb [17-19].

Tae-Wook Chun from the National Institute of Allergy and Infectious Diseases, USA, offered a sobering case report highlighting the difficulty of addressing HIV persistence. Chun described an individual who had been on suppressive ART for 10.5 years and in whom quantitative co-culture could only detect one infected cell out of 1.6 billion CD4 T cells, the lowest level Chun has ever seen. But when ART was stopped, there was only a 40-day delay in viral rebound. Chun went on to argue that persistent, ongoing HIV replication in sanctuary sites like the gut (as indicated in Joseph Wong’s study) may have a greater role in sustaining viral reservoirs than is currently assumed.

The first day of the workshop closed with a discussion of an increasingly famous but anonymous individual who appears to have been functionally cured of HIV infection. Haematologist Gero Hütter provided an update on the published case report [20]; after three years of follow up without ART, HIV can still not be detected in blood or tissues, and the CD4 count has reached the highest level since the original diagnosis. Because the individual’s treatment involved a bone marrow transplant necessitated by acute myelogenous leukemia, there was widespread agreement that the result cannot easily be translated into a therapeutic approach for broader use. However, it is being viewed as a valuable proof of concept that a cure for HIV infection is achievable.

### What is the role of the immune system in HIV persistence?

Brigitte Autran from the Université Pierre et Marie Curie in Paris, France, outlined what she described as the yang (bad) and yin (good) of HIV-specific immune responses, noting that in some circumstances, HIV-specific CD4 T cells can become targets for virus infection, while in others these responses are associated with immunological control of HIV replication. Autran highlighted work by Benjamin Descours (presented later in the same session) showing that among individuals with the protective HLA alleles, B*27 and B*57, Gag-specific CD8 T cell responses correlate negatively with the size of the HIV reservoir in central memory CD4 T cells. Autran is involved in two studies, Eramune 01 & 02, which will evaluate whether the addition of therapeutic vaccination or the cytokine IL-7 to intensified ART can reduce the size of the HIV reservoir.

Five oral abstract presentations covered different aspects of the interaction between the immune system and HIV persistence. Vicente Planelles from the University of Utah, USA showed data supporting the suggestion by Nicolas Chomont [11] that memory CD4 T cells containing HIV DNA can undergo homeostatic proliferation and thus increase the number of latently infected cells [21]. Based on a suite of *in vitro* studies, Vanessa Evans from Monash University in Victoria, Australia, made the case that myeloid dendritic cells can induce HIV latency in non-proliferating CD4 T cells [22]. Mathias Lichterfeld from Massachusetts General Hospital in Boston, USA, presented new data suggesting that the CD4 T cells of elite controllers may be better able to resist HIV infection due to greater expression of a host protein, called p21.

### What host factors are at play?

Paul de Bakker from Harvard University, USA, gave an introduction to genetics studies and the role that they can play in HIV research. Approaches vary depending on the frequency of a given genetic variation in the population. To date, study results have identified several consistent associations with control of HIV replication within the MHC region on chromosome six of the human genome, but no other novel gene candidates have emerged. De Bakker cited a recently published analysis of 2500 HIV-positive individuals that failed to replicate many of the isolated reports of genetic associations that have appeared in the literature [23]. Future studies will need large, diverse cohorts of individuals with well-defined phenotypes, de Bakker suggested.

Oral abstract presentations focused on host cell proteins involved in viral replication, including Tetherin [24], rhTRIM5α [25] and LEDGF/p75 [26]. Claudio Casoli from Università degli Studi di Milano, Milan, Italy, also presented results suggesting that HIV infection modulates expression of host cell micro-RNAs [27].

### What are potential therapeutic interventions and how to evaluate them?

The task of giving an overview of therapeutic issues fell to Frank Maldarelli from the National Cancer Institute, USA. Maldarelli described the “single copy” assay developed by Sarah Palmer that can measure down to one copy of HIV RNA in three milliliters of plasma. Maldarelli’s group has used the assay to show that there is persistent low-level viremia in most individuals on treatment that is independent of the specific antiretroviral regimen they are using. The level of persistent viremia is stable over long-term follow up, and is correlated with pre-treatment viral load. Maldarelli echoed Steve Deeks’s review of the results of ART intensification studies, noting that most had shown no effect. The critical test of any protocol aiming for HIV eradication, Maldarelli argued, will be to monitor what happens after ART is interrupted.

Among the oral abstract presentations, Carolina Garrido from Hospital Carlos III, Madrid, Spain, reported an unexpected impact of the integrase inhibitor raltegravir on thymopoiesis (the production of new naïve T cells by the thymus) [28]. The mechanism for the effect is unclear, although there is a published report that integrase inhibition could impact T cell gene rearrangements that occur in the thymus due to interference with RAG1/2 recombinase [29].

Carolina Gutiérrez from Hospital Ramón y Cajal, Madrid, Spain, presented a very small, nine-person study of ART intensification with the CCR5 inhibitor maraviroc. The results suggested some reduction in the number of HIV-infected cells; however, there was also an unexpected increase in 2-LTR circular DNA and HIV RNA (measured by the Sarah Palmer single copy assay) in some participants [30]. Larger studies will be needed to confirm these findings.

**Figure 2:**
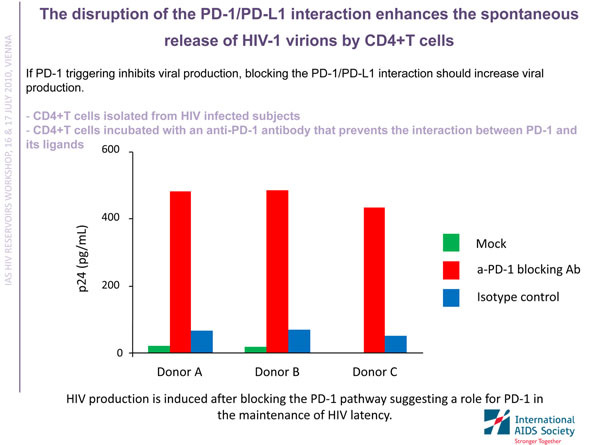
**Prodding HIV out of latency via PD-1**. Sandrina Da Fonseca showed that blocking the PD-1 pathway induced HIV production by latently infected CD4 T cells, suggesting PD-1 inhibition could contribute to the depletion of the HIV reservoir. This slide was presented by Sandrina Da Fonseca at the workshop “Towards a Cure: HIV Reservoirs and Strategies to Control Them”.

Sandrina Da Fonseca from the Vaccine and Gene Therapy Institute, Florida, USA, outlined the potential utility of a cellular marker, named PD-1, in identifying memory CD4 T cells harbouring HIV. Da Fonseca also showed that inhibiting PD-1 may awaken the latent viral genomes in these cells, potentially offering a new method for depleting the HIV reservoir (Figure 2) [31].

**Figure 3:**
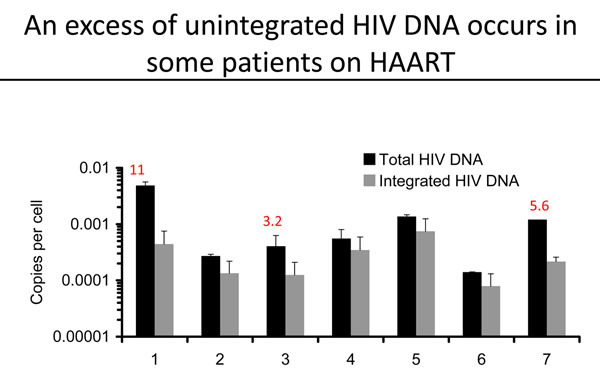
**Flickering embers of infection.** Una O’Doherty reported that some individuals on HAART show sporadic excesses of non-integrated HIV DNA, suggestive of intermittent episodes of viral replication. This slide was presented by Una O’Doherty at the workshop “Towards a Cure: HIV Reservoirs and Strategies to Control Them”.

Una O’Doherty from the University of Pennsylvania, USA, revealed a new and potentially important method for the measurement of residual viral replication in individuals on ART. The approach compares total levels of HIV DNA with the amount of integrated HIV DNA in order to quantify non-integrated proviral DNA. O’Doherty showed that around two-thirds of people on suppressive ART sporadically display an excess of non-integrated proviral DNA, strongly suggesting that viral replication cycles sometimes occur despite ART (Figure 3) [32].

Switching to the SIV/macaque model, Andrea Savarino from Istituto Superiore di Sanità, Rome, Italy, studied the gold-based compound auranofin (Gar1041) which induces downregulation of the CD27 molecule on central memory CD4 T cells, thereby potentially reducing the latent HIV reservoir in this cell type. Savarino observed a significant decay in proviral SIV DNA when Gar1041 was administered to macaques infected with SIVmac251 and treated with intensified ART. There was also evidence of delayed viral rebound and improved immune control of SIV replication when ART was stopped [33].

### Closing session – eradication versus remission: is eradication possible?

The workshop closed with two presentations addressing broader issues related to the search for a cure. Sharon Lewin from Monash University in Victoria, Australia, provided context on the limitations of ART, citing data from the Danish HIV cohort showing that, while vastly improved, life expectancy for HIV-positive individuals has yet to reach that of their HIV-negative counterparts [34]. ART is also encumbered by side effects that can significantly impinge on an individual’s quality of life, particularly over the long term.

Lewin also pointed out that because the number of new HIV infections continues to outpace the number of people starting ART by 5:2, the resources needed to treat everyone will eventually become impossibly gargantuan. Lewin spoke eloquently of the need to engage affected communities in HIV persistence research, highlighting the invasive procedures required by some studies and emphasizing the importance of translating scientific language into more accessible terms. Finding a cure for HIV infection, Lewin concluded, is a human rights issue.

Daria Hazuda from Merck & Co, Inc, USA, provided a perspective from the pharmaceutical industry, offering insights into the daunting logistical challenges of the drug discovery process. Hazuda cited a 2003 study estimating that for every 5000 to 10,000 compounds synthesized for *in vitro* evaluations, approximately 250 are selected for further preclinical investigation in cellular and animal models. Of these, an average of five compounds move into clinical trials and only one obtains FDA approval [35].

Hazuda noted that the search for drugs aiming to reverse HIV latency offers additional challenges as there are a variety of cellular models available and results may not always be compatible from one system to the next. Merck has developed a high throughput assay to identify compounds that can activate latent HIV, focusing particularly on histone deacetylase (HDAC) inhibitors. Hazuda also described the potential advantages of synergy between compounds, such as HDAC inhibitors and protein kinase C (PKC) activators (e.g., prostatin). Hazuda cited an unpublished study by Paul Luciw that found that this combination could reduce tissue levels of viral DNA in ART-treated macaques infected with a simian immunodeficiency virus containing HIV reverse-transcriptase (SHIV-RT); however, there was no delay in viral rebound when ART was interrupted.

Hazuda concluded with some questions that need to be addressed for the field to move forward, such as which animal model is best, what criteria will be used to justify human trials of new approaches, and the indicators that might be used to trigger the interruption of ART in order to judge whether an approach has worked.

## Conclusions

“Towards a Cure: HIV Reservoirs and Strategies to Control Them” offered a preliminary mapping of the difficult terrain that must be navigated if a cure for HIV infection is to be achieved. Further work is clearly required to provide a more detailed topography, as certain fundamental issues, such as the possible contribution of localized viral replication to persistence and the exact nature of viral sanctuary sites, remain to be clarified.

The importance of translational clinical research involving potential therapeutic approaches emerged as a key theme, as this work can shed light on the uncertainties that still lurk amid the basic science. Workshop chair and International AIDS Society (IAS) President-Elect Françoise Barré-Sinoussi made it clear that the topic is a key priority for the IAS, and the dialogue and exchange of ideas begun in Vienna will continue until the journey towards a cure is completed.

## Competing interests

RJJ is an employee of the Treatment Action Group (TAG), a 501(c)(3) non-profit community-based HIV/AIDS advocacy organization. TAG was a co-sponsor of the workshop.

## Author contribution

RJJ wrote the article and approved the final version.
